# Ant Mimicry by an Aphid Parasitoid, *Lysiphlebus fabarum*

**DOI:** 10.1673/031.010.12601

**Published:** 2010-08-06

**Authors:** Arash Rasekh, J.P. Michaud, Aziz Kharazi-Pakdel, Hossein Allahyari

**Affiliations:** ^1^ Department of Plant Protection, College of Agriculture, University of Tehran, Karaj, Iran; ^2^ Kansas State University, Agricultural Research Center — Hays, 1232 240^th^ Ave, Hays, KS, 67601, 785-625-3425

**Keywords:** *Aphis fabae*, aphid defense, feeding, foraging, honeydew, host handling, host recognition, oviposition, parasitism, *Vicia fabae*

## Abstract

In Iran, *Lysiphlebus fabarum* (Marshall) (Hymenoptera: Braconidae: Aphidiinae) is a uniparental parasitoid of the black bean aphid, *Aphis fabae* Scopoli (Hemiptera: Aphididae), that possesses various highly evolved adaptations for foraging within ant-tended aphid colonies. Direct observations and video recordings were used to analyze the behavior of individual females foraging for *A. fabae* on bean leaf disks in open arenas in the laboratory. Females exploited aphids as hosts and as a source of food, allocating within-patch time as follows: resting - 10.4%, grooming - 8.2%, searching - 11.5%, antennation (host recognition) - 7.5%, antennation (honeydew solicitation mimicking ants) - 31.9%, abdominal bending (attack preparation) 19.7%, probing with the ovipositor (attack) - 10.8%. The mean handling time for each aphid encountered was 2.0 ± 0.5 min. Females encountered an average of 47.4 ± 6.4 aphids per hour, but laid only 1.2 eggs per hour. The ovipositor insertion time for parasitism ranged from 2 sec to longer than a minute, but most insertions did not result in an egg being laid. *A. fabae* defensive behaviors included kicking, raising and swiveling the body, and attempts to smear the attacker with cornicle secretions, sometimes with lethal results. Food deprivation for 4–6 h prior to testing increased the frequency of ant mimcry by *L. fabarum*. Females also used ant-like antennation to reduce *A. fabae* defensive behavior, e.g. the frequency of kicking. *L. fabarum* attacks primed *A. fabae* to be more responsive to subsequent honeydew solicitation, such that experienced females improved their feeding success by alternating between the roles of parasitoid and ant mimic. These results reveal the possibility for mutualisms to evolve between *L. fabarum* and the ant species that tend *A. fabae*, since *L. fabarum* receive ant protection for their progeny and may benefit the ants by improving *A. fabae* responsiveness to honeydew solicitation.

## Introduction

Aphid defensive behavior and attendance by ants are perhaps two of the most important forces driving the evolution of foraging behavior in aphid parasitoids (Völkl and Mackauer 2000). The black bean aphid, *Aphis fabae* Scopoli (Hemiptera: Aphidiinae), is a major pest of sugar beet, *Beta vulgaris* and broad bean, *Vicia faba* L., (Völkl and Stechmann 1998; Nuessly et al. 2004). *Lysiphlebus* fabarum (Marshall) (Hymenoptera: Braconidae) is one of the most abundant parasitoids of *A. fabae* in agroecosystems (Stary 1970). *L. fabarum* has mainly thelytokous reproduction in central Europe (Nemec and Stary 1985; Stary 1986) and attacks *A. fabae* on various crops and weeds (Völkl and Stechmann 1998; Raymond et al. 2000). Ant attendance is thought to protect *A. fabae* colonies against many natural enemies (Dixon and Agarwala 1999; Völkl et al. 2007; Kunert et al. 2008). However, *L. fabarum* may benefit from ant attendance and parasitize *A. fabae* at higher rates in their presence. Foraging *L. fabarum* females have been observed to remain longer and parasitize more aphids in ant-tended colonies than in unattended colonies (Völkl and Stechmann 1998). To this end, the parasitoid appears to possess specific adaptations, chemical and behavioral, that generally negate the aggressive responses of various ant species including *Lasius niger, Lasius fuliginosus, Myrmica* spp., and *Formica polyctena* (Völkl and Mackauer 1993; Völkl 1997).

Aside from recruiting ants, aphids may utilize a variety of behaviors to directly defend themselves from parasitoid attacks (Völkl and Kroupa 1997), though these are not always effective (Wyckhuys et al. 2008; Desneux et al. 2009). Some species (e.g. *Acyrthosiphum pisum*) may escape by simply walking away (Weisser 1994; Walker and Hoy 2003) or quickly dropping from the plant (Chau and Mackauer 1997; Villagra et al. 2002), even though such behaviors are not without cost (Dill et al. 1990). However, species such as *A. fabae* feed with their stylets so deeply imbedded in plant tissues that quick release of the plant is often impossible. Consequently, *A. fabae* deploys a range of alternative tactics that include raising and swiveling the body, kicking, and efforts to smear the attacker with cornicle secretions, all of which can substantially increase host handling time for parasitoids. Aphid cornicle secretions are composed largely of triglycerides (Callow et al. 1973) and resemble a fast-drying liquid wax with strongly adhesive properties. Droplets of cornicle secretion can entrap parasitoids and seal the mouthparts of predatory larvae (Butler and O'Neil 2006), thus posing a significant hazard for smaller natural enemies.

*L. fabarum* is perhaps unique among the Aphidiinae in soliciting honeydew directly from aphids. Consequently, aphids serve both as hosts and as a source of food for this species, a situation analogous to host feeding in which parasitoids consume tissues and hemolymph of some hosts and oviposit in others (e.g., Takada and Tokumaku 1996). Host feeding has been reported from 17 different families of parasitic Hymenoptera (Jervis and Kidd 1986), but honeydew feeding differs significantly from host feeding in several important respects. It does not cause any host mortality, nor does it provide any protein for egg maturation, but it does require the cooperation of the host. In these experiments, individual *L. fabarum* females were released in the laboratory onto bean leaf disks infested with *A. fabae* in the laboratory.

Continuous observations and video recordings were made, females' proportional time allocation to various activities was measured, and the functions of different behaviors with respect to feeding and parasitism were inferred.

## Materials and Methods

### Insect colonies

A thelytokous population of *L. fabarum* was established from mummies collected from *A. fabae* colonies feeding on broad bean in a field in Zanjan Province, Iran, in June 2007. A stock colony of *A. fabae* was maintained on potted broad bean, *V. faba* var. Sarakhsi, grown in pots filled with fertilized sawdust in growth chambers at 20 ± 1° C, 65–75% RH, and a 16:8 L:D photoperiod. *L. fabarum* was reared on *A. fabae* fed on broad bean under the same conditions. All *A. fabae* used in experiments were four days (± 6 h) old at 20° C (late second to early third instar nymphs). Synchronous cohorts of *L. fabarum* were produced by exposing second instar *A. fabae* to three-day-old female *L. fabarum* in a ventilated plastic cylinder (8.0 cm diameter × 20.0 cm) for a period of six h and then transferring the *A. fabae* to potted bean plants in a growth chamber until those parasitized formed mummies. Mummies were carefully removed from plants and isolated in gelatin capsules (vol. = 0.95 cm^3^) until emergence, whereupon each adult female was released into her own ventilated plastic cylinder (3.5 cm diameter × 7.0 cm) and provisioned with diluted honey (as droplets on a strip of wax paper) and water (on a cotton roll). The water was refreshed daily and the diluted honey was refreshed every second day. All females were used in experiments when they were 72 ± 4 h of age without prior exposure to aphids. All experiments were carried out in a growth chamber under the same physical conditions.

### Longevity

A synchronous cohort of wasps was produced by exposing second instar *A. fabae* to three-day-old female *L. fabarum* (as above) and then transferring the *A. fabae* to potted bean plants. Following emergence in individual gelatin capsules, a total of 28 females were isolated in ventilated cylinders and provisioned with water and diluted honey as above. Females were examined every 12 hours, and mortality was recorded until all females were dead.

### Oviposition threshold

The objective of this experiment was to determine a threshold ovipositor insertion time that would distinguish successful attacks in which an egg was laid in a host from mere investigative probing. In each replication (*n* = 10) a female *L. fabarum* was released into a glass Petri dish (3.5 cm diameter × 1 cm) containing a leaf disk of broad bean on which 30 second-instar *A. fabae* had been permitted to settle several hours earlier. Each female was permitted to attack 12 *A. fabae* only once. The duration of ovipositor insertion was recorded for each attack and the *A. fabae* was then promptly removed from the arena. Each attacked *A. fabae* was reared individually in a plastic Petri dish (6 cm diameter × 1 cm) containing a broad bean leaf on 1.5% agar. After four days in a growth chamber (as above) the aphids were dissected to verify the presence or absence of *L. fabarum* larvae. The data were analyzed by one-way ANOVA.

### Foraging observations

The objective of these observations was to quantify time allocation by *L. fabarum* females to various within-patch behaviors. These were categorized as follows: resting, grooming, searching the leaf surface, host antennation (three forms), abdominal bending associated with visual host examination in preparation for attack, and probing (insertion of the ovipositor).

Female *L. fabarum* (*n* = 20) were introduced singly into Petri dish patches (as above) that each contained 15 second-instar *A. fabae* that had been allowed to settle and feed on a leaf disk of *V. faba*. The exact positions of all aphids on the leaf disk were mapped on a piece of paper so that all *A. fabae* probed by the female could be tracked. Once a female encountered the first *A. fabae*, the lid of the arena was removed to create an open patch and a stopwatch was started. When the parasitoid walked out over the edge of the dish, the watch was stopped and patch residence time was recorded. Active foraging time was defined as the total time spent on the patch minus the time spent cleaning or resting. Each female was observed continuously under a stereomicroscope while she remained in the patch and the time of onset and duration of all distinguishable behavioral events were recorded using an MP3 voice recorder. The audio recordings were subsequently transcribed and used to determine the proportional time allocation by each female to each type of behavior while within the patch.

In order to estimate rates of parasitism, a subset of the aphids attacked by each female was removed from the arena for rearing. Only aphids receiving ovipositor probes ≥ 25 sec in duration were removed, since many attacks were brief and repeated, and since we also wished to observe the responses of previously attacked aphids. Each such cohort of aphids attacked by a female was placed on an excised bean shoot in a mini-cage on a small container of water. All aphids probed < 25 sec were left in the arena but had their positions mapped so they could be distinguished from previously unprobed aphids. After four days in a growth chamber, all attacked *A. fabae* from each replicate were dissected, and the larvae within them were counted.

Mean aphid handling time was calculated for each female as the total time spent addressing *A. fabae* divided by the number of *A. fabae* encountered (antennation + abdominal bending + probing / no. *A. fabae* encounters). Mean host handling time was calculated specifically for *A. fabae* that were actually probed, regardless of the duration (antennation + abdominal bending + probing / no. aphids probed) was also made. The correlation between solicitation antennation events and *A. fabae* kicking events was analyzed using Pearson's Correlation Coefficient. Secretion of honeydew droplets by previously probed vs. unprobed *A. fabae* was analyzed with a paired *t*-test.

### Food deprivation

Having observed females solicit and receive honeydew from *A. fabae*, it was realized that *L. fabarum* females utilized the aphid colony as a food resource as well as a host patch, and that a female's hunger level might influence her time allocation to various behaviors while in a patch. To solicit honeydew, females utilized a distinct form of antennation that appeared to mimic aphid-tending ants. It was reasoned that this behavior should be expressed more often by hungry females than by satiated ones if its purpose was to obtain food. In the third experiment, experimental females (*n* = 24) were randomly divided into two groups, one provided diluted honey *ad libitum* and the other starved for a period of 4– 6 hours before testing. Each female was then introduced into a patch (as above) with 15-second-instar aphids. Once a female encountered the first aphid, her behavior was recorded continuously for five minutes by direct observation under a stereomicroscope, and honeydew solicitation events were tallied. The data were analyzed using Fisher's exact test.

### Video recording

In order to obtain video clips of the various behavioral interactions between female parasitoids and aphids, a series of females (*n* = 60) were introduced individually into patches containing aphid-infested leaf sections taken from the stock *A. fabae* colony. Behavioral events were recorded using a NIKON 6 megapixel digital video camera mounted on a stereomicroscope. The resulting video streams were edited using iMovieVideo® software on an iMac® computer (Apple, Inc., www.apple.com) and exported as Quicktime® files.

## Results

### Longevity

The median longevity of wasps with *ad libitum* access to dilute honey and water under the experimental conditions was 4.5 days; the mean was 6.3 days. Fourteen of the 28 wasps (50%) died in their 7^th^ day of life, and 4 remained alive on day 8.

**Figure 1.**
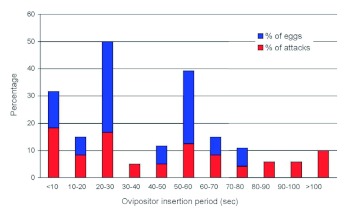
Proportional distribution of ovipositor insertion times and eggs laid for 120 attacks by *Lysiphlebus fabarum* on *Aphis fabae* (10 females each attacking 12 second instar nymphs). The overall mean ovipositor insertion time was 51.0 sec, compared to 38.7 sec for attacks that resulted in oviposition (15). High quality figures and videos are available online.

### Oviposition threshold

The mean ovipositor insertion time (*n* = 120 aphids attacked) was 50.5 ± 5.9 sec and only 15 *A. fabae* in total (12.5%) were parasitized. There was no significant variation among females in ovipositor insertion time (*F* = 1.83; df = 9, 110; p = 0.071) or in the number of *A. fabae* parasitized (*F* = 1.43; df = 9, 110; p = 0.184). The mean duration of ovipositor insertions resulting in parasitism was 38.7 ± 6.1 sec, but no clear threshold time was evident (Figure 1). Insertion times as long as 385 s failed to result in parasitism, and in two cases, successful parasitism occurred with an insertion of only 2 sec. Only 3 of the 40 *A. fabae* probed < 25 sec (7.5%) were parasitized.

### Parasitoid behaviors

Three distinct types of host antennation behavior were distinguishable: recognition antennation, solicitation antennation, and oviposition antennation. Recognition antennation was employed during host searching and appeared to confirm host recognition (as determined by arrestment of movement) when *A. fabae* was encountered. The antennae were held in a straight configuration and tapped the aphid quickly several times, usually for a period of less than 5 sec. Host recognition appeared to be cued by antennal contact with the host cuticle, and females were observed to use ovipositor probes to investigate shed *A. fabae* skins and mummified *A. fabae* following antennal contact with them.

Solicitation antennation was used to elicit honeydew and involved lightly and repeatedly tapping *A. fabae* with the end of the antennae curved downwards in the manner of a tending ant (Video 1). Solicitation antennation by inexperienced females often took a long time to result in a reward, sometimes as long as 20– 30 minutes. Droplets of honeydew presented to *L. fabarum* were either eaten directly from the aphid's anus or immediately from the surface of the leaf. The latter behavior often occurred when a droplet became stuck to a female's appendage and was subsequently transferred to the leaf. Although *A. fabae* are able to discard honeydew droplets by kicking them away with a quick flick of a hind tarsus (Video 2), droplets secreted in response to solicitation were quickly withdrawn back into the body of the *A. fabae* if they were not discovered by the female within a few seconds (Video 3). The 20 females tested succeeded in soliciting a total of 51 droplets of honeydew during the course of the experiment, and they drank 35 of them, 22 directly from the anus of the aphid, and 13 following their displacement onto the leaf surface, whereas six were withdrawn by the *A. fabae*. Hungry females were more likely to elicit fresh honeydew directly from aphids and only resorted to licking honeydew from the leaf surface when solicitation efforts were unsuccessful. More droplets of honeydew were obtained from *A. fabae* that were previously probed with the ovipositor than from those that were previously unattacked (χ^2^ = 4.01, p < 0.05).

**Video 1.**
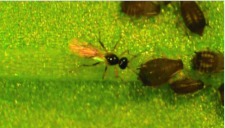
Antennation behavior of *Lysiphlebus fabarum* during solicitation of honeydew from *Aphis fabae*. High quality figures and videos are available online.

**Video 2.**
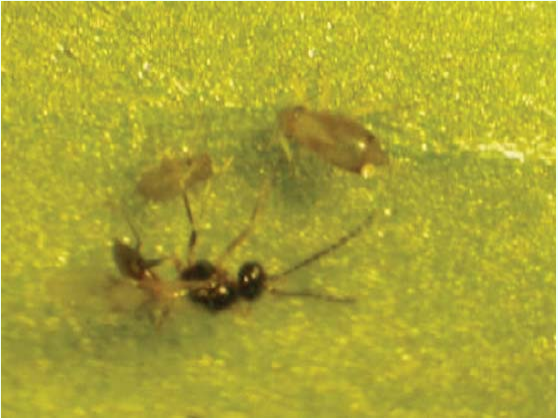
An *Aphis fabae* nymph kicks away a droplet of honeydew using its hind tarsus. High quality figures and videos are available online.

**Video 3.**
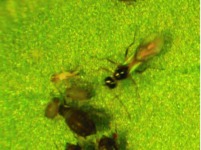
An *Aphis fabae* nymph withdraws a droplet of honeydew when it is overlooked by an *L. fabarum* female following presentation. High quality figures and videos are available online.

Attack antennation was observed specifically during ovipositor insertion and took the form of a gentle stroking of the dorsal surface of *A. fabae* with the antennae held in a straight orientation (Video 4). The function of attack antennation appeared to be the suppression of *A. fabae* defensive behavior during ovipositor insertion, although it did not mimic ant behavior.

In addition to other familiar parasitoid behaviors such as searching, resting, and grooming, female *L. fabarum* adopted a characteristic posture prior to host attack that was termed ‘abdominal bending.’ In this behavior, the wasp stood at a relatively fixed distance from its host with the antennae held vertically at right angles and appeared to examine the aphid visually while curving the abdomen forward in preparation for a strike with the ovipositor. Females often abandoned *A. fabae* following a series of brief strikes, only to return to it subsequently and solicit honeydew, as if alternating between the role of parasitoid and ant.

As previously reported by Völkl and Stary (1988), aggregations of females more or less continuously interacting with one another on the ceilings of cages and rearing containers were often observed. These interactions involved antennation, abdominal bending, and probing with the ovipositor. The probing behavior appeared investigative rather than aggressive and did not result in any overt injuries, nor did it appear to repel the recipients or induce their dispersal. It seems probable that the same cuticular elements of host mimicry that serve to camouflage the wasp as ants are also sufficient to confuse conspecific females. Females were also observed investigating their own mummy by ovipositor probing within a minute or so of emergence.

**Video 4.**
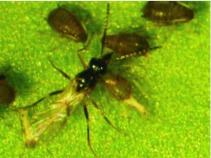
A female *Lysiphlebus fabarum* strokes an *Aphis fabae* nymph with its antennae during ovipositor insertion. High quality figures and videos are available online.

### Time allocation

**Figure 2.**
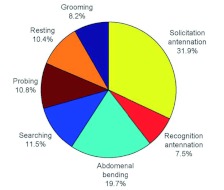
Proportional time allocation of three-day-old *Lysiphlebus fabarum* females to various behaviors while foraging alone in open patches consisting of 15 second instar *Aphis fabae* feeding on a bean leaf disk in a Petri dish. High quality figures and videos are available online.

*L. fabarum* females appeared very active within host patches, yielding a total of 2691 behavioral events during 24 hours of observation, including 894 aphid encounters. Females usually did not start to forage immediately upon release into a host patch, and the average time interval between release and encounter with the first aphid was 2.7 ± 0.6 min. Once aware of the presence of *A. fabae*, females averaged 72.1 ± 5.6 min within the patch, actively foraged for a mean of 58.9 ± 4.9 min, encountered a mean of 47.4 ± 6.4 aphids per h of active foraging, made an average of 14.2 ± 2.1 ovipositor probes, and parasitized a mean 0.65 ± 0.27 aphids each. The mean handling time for aphids encountered was 1.98 ± 0.53 min, but this was reduced to 1.51 ± 0.13 min when only probed *A. fabae* were considered. A total of 13 out of 100 aphids probed ≥ 25 sec were parasitized. Assuming a parasitism rate of 7.5% for aphids probed < 25 sec (based on results of the oviposition threshold experiment), the mean oviposition rate was 1.2 eggs/hour of active foraging, or slightly less than one egg laid for every ten aphids encountered. The mean (± SEM) amount of time spent on each distinct behavior was: antennation, 27.8 ± 4.5 min (solicitation = 22.5 ± 3.6 min, host recognition = 5.3 ± 0.9 min, and attack = 0.85 ± 0.17 min); abdominal bending, 13.9 ± 2.0 min; searching, 8.1 ± 0.9 min; probing with the ovipositor, 7.6 ± 1.2 min; resting, 7.3 ± 1.7 min; and grooming 5.8 ± 3.4 min. Mean proportional patch time allocation is depicted in Figure 2 with attack antennation excluded because it occurred during probing.

### Aphid defensive behavior

Although *A. fabae* typically remained anchored to plant tissues by their stylets when attacked by female *L. fabarum*, they often struggled violently. Their various defensive behaviors (Video 5) included kicking (56 events), raising and swiveling the body (4 events), withdrawing the stylet and escaping (8 events), and producing a droplet of cornicle secretion (12 events). When female *L. fabarum* contacted a droplet of cornicle secretion (7 events), they either spent an average of 10.2 minutes thereafter in grooming behavior or became permanently stuck to the aphid, typically resulting in death of the parasitoid (Video 6). However, females appeared to utilize solicitation antennation to diminish defensive responses, ostensibly deceiving the aphids into mistaking them for ants. For example, there was a significant negative correlation between the time spent in solicitation antennation and the number of kicking events (Pearson Correlation Coefficient = - 0.39, < 0.05).

**Video 5.**
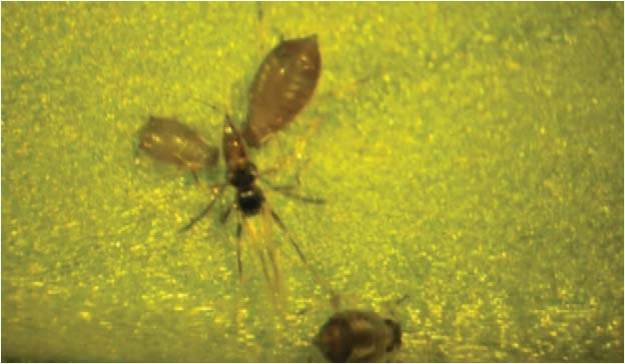
Defensive behaviors of *Aphis fabae* in response to probing by *Lysiphlebus fabarum*: Swiveling, kicking, attempting to smear attacker with cornicle secretions. High quality figures and videos are available online.

### Food deprivation

Hungry females exhibited solicitation antennation more often than did satiated females (χ^2^ = 7.11, p < 0.01). Eleven of the 12 females (92%) deprived of food for 4–6 h prior to testing displayed solicitation antennation behavior when provided with a patch of aphids, compared to only 3 (25%) of the females provided with continuous access to diluted honey.

## Discussion

This particular strain of *L. fabarum* exploited *A. fabae* as a source of both food and hosts, and the time females allocated to honeydew solicitation was affected by the females' hunger level. Although honeydew feeding by parasitoids is a well-recognized phenomenon (Jervis and Kidd 1986), to the authors' knowledge this is the first report of a braconid wasp soliciting honeydew directly from aphids. However, several parasitoid species specializing on root-feeding aphids are known to have evolved close relationships with the ants that tend them (Takada and Hashimoto 1985). For example, *Paralipsis enervis* is a species in which females use a combination of chemical and behavioral mimicry not only to avert aggression by *L. niger* workers, but also to obtain food from them via trophallaxis (Völkl et al. 1996). One benefit of direct honeydew solicitation is the acquisition of material with a higher water content and lower viscosity, relative to residues available on the leaf surface. Viscosity is known to be a factor limiting the nutritional value of honeydew to parasitoids (Faria et al. 2008), and the ability to obtain it directly from aphids could provide a critical advantage in desiccating environments. On the other hand, obtaining fresh honeydew entails a substantial cost in terms of the time wasps spend in solicitation behavior, and there is recent evidence to suggest honeydew may represent a relatively inferior food relative to other natural sugar sources (Wackers 2008, Wyckhuys 2008b).

**Video 6.**
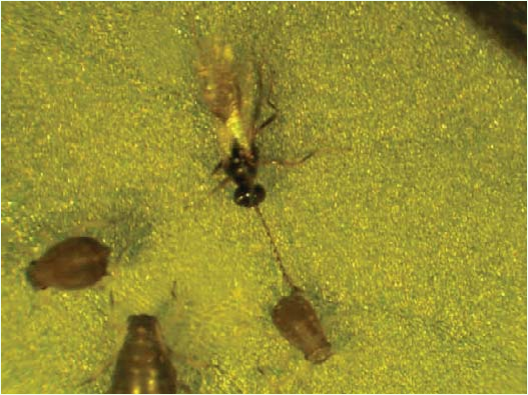
Death of a female *Lysiphlebus fabarum* following adhesion of one antenna to a droplet of *Aphis fabae* cornicle secretion (condensed sequence). High quality figures and videos are available online.

These observations suggest that these wasps deceive *A. fabae* into responding to them as if they were ants. Although honeydew is a waste product for the aphids, it acquires value as a reward in the presence of tending ants that offer protection from natural enemies. In the absence of tending insects, *A. fabae* were quite able to dispose of honeydew by flicking the droplet away with a hind tarsus, but they never did so when being solicited by *L. fabarum*. Rather, they withdrew and conserved any droplet that was not consumed, consistent with valuation of the honeydew as a resource. The solicitation behavior of *L. fabarum* overtly resembled the antennal drumming used by ants to solicit honeydew. Suppression of defensive responses toward parasitoids in the presence of tending ants is known for *A. fabae* and other aphid species (Völkl 1997), but *L. fabarum* effectively used ant mimicry to diminish aphid defensive reactions in their absence, most notably the frequency of kicking. The death of several *L. fabarum* during the observation period due to smearing with cornicle secretions highlights the hazards of handling *A. fabae* (Wynn and Boudreaux 1972) and the value of suppressing their defensive responses.

Although *L. fabarum* is a strongly proovigenic parasitoid that emerges with many hundreds of mature eggs (Belshaw and Quicke 2003) and lives for only a few days, females may at times become egg-limited while foraging, not unlike other synovigenic parasitoids (e.g. Heimpel and Collier 1996). Various authors have modeled the consequences for time-limited parasitoids of partitioning effort between seeking food versus seeking hosts when these occur in separate patches (Sirot and Bernstein 1996, Tenhumberg et al. 2006, and references therein), so the ability to obtain both resources in the same patch may be construed as adaptation for time conservation, just as thelytoky eliminates the need to allocate time for mate searching. However, once within a patch, *L. fabarum* females did not appear to make oviposition a priority and laid only one egg per hour of foraging time. This is an exceptionally low value considering that *Lysiphlebus* spp. typically make anywhere from 6 to > 40 ovipositions per h even in the absence of ants that may render their foraging more efficient by virtue of reducing aphid defensive reactions (Völkl 1997). Females spent a full third of their time soliciting honeydew, and *A. fabae* often appeared reluctant to respond. Aphids could conceivably utilize reticence to induce ants to spend longer periods within the colony, although their low responsiveness could also be partly an artifact of the absence of real ants in these experiments. Honeydew excretion rates may have been reduced because the leaf discs represented an inferior food source for *A. fabae* compared to intact plants, but even *A. fabae* with honeydew available did not relinquish it without an extended period of solicitation. Furthermore, females spent an additional 30% of their time examining and probing *A. fabae*, apparently without ovipositing, despite being able to lay an egg in a two second insertion. The ovipositor is a complex sensory organ with a diversity of mechanosensory and gustatory sensillae (Larocca et al. 2007) that play an important role in assessing host suitability. However, the tendency of females to probe their own mummies and one another further supports an interpretation of ovipositor probing as an investigatory behavior in other contexts. Whereas recognition antennation appeared to rapidly confirm host identity, subsequent abdominal bending prior to attack was often associated with an extended period of apparent visual examination. Some movement on the part of the aphid was normally required to elicit a strike, as previously noted for other aphidiid species (Michaud and Mackauer 1994a, 1994b). However, oviposition decisions in this species are largely determined by chemical cues encountered during ovipositor probing (Hildebrands et al. 1997).

Various *Lysiphlebus* species possess cuticular lipids and hydrocarbons similar to those of their aphid hosts that function in providing them with a generalized immunity from ant aggression (Liepert and Dettner 1993, 1996). With this chemical camouflage, *L. fabarum* enjoys reduced predator interference while foraging in ant-tended colonies (Völkl and Stechmann 1998) and higher rates of offspring survival (Stary 1987, Meyerhofer and Klug 2002). This chemical camouflage is so convincing that females antennating conspecifics investigate further with ovipositor probing. The apparent low rate of host parasitism by *L. fabarum* is of particular interest because aphidiid species specializing on ant-tended resources tend to have exceptionally high rates of parasitism (Völkl 1997). However, thelytokous *L. fabarum* females avoid the ‘cost of meiosis’ associated with producing sons (Maynard Smith 1978), and they produce twice as many daughters as an arrhenotokous female parasitizing the same number of hosts. Secondly, specialization on ant-tended aphids may further reduce the number of hosts required to ensure a minimum level of reproductive success. Intraguild predation and hyperparasitism are both major sources of mortality for immature aphid parasitoids that may be eliminated by ant attendance (Mackauer and Völkl 1993, Hübner and Völkl 1996, Hübner 2000, Völkl and Sullivan 2000, Kaneko 2004), such that females may gain more fitness by enlisting ants to ensure the survival of a few progeny, than by attempting to produce a large number without such protection.

Avoidance of self-superparasitim (Rosenheim and Mangel 1994) seems an unlikely explanation for the low oviposition rate of this *L. fabarum* strain, since many more *A. fabae* could be parasitized in each patch before self-superparasitism would become a significant risk. Spreading offspring among patches to avoid risk (Ayal and Green 1993, Cronin and Strong 1993) seems a more plausible possibility. Additional observations of wasps in ant-tended colonies in the field would be useful to determine whether oviposition rates change in the presence of ants. The exploitation of mutualisms by ‘third parties’ is a relatively common ecological phenomenon and ant mutualisms are particularly vulnerable to exploitation and cheating (Soberon Mainero and Martinez del Rio 1985; Bronstein 2001). However, by attacking many more *A. fabae* than they parasitize, *L. fabarum* females increase responsiveness to honeydew solicitation, an effect that should also benefit tending ants by reducing the effort they must expend in soliciting honeydew. In this context, some host probing may constitute harassment for purposes of improving the food supply, both for ants and for the parasitoids themselves. Mutualistic interactions between wasp and ant are not depicted in the range of possible interactions described by Völkl (1997), but could be evolutionarily stable provided the benefits of the primary ant-aphid mutualism are conserved, i.e. the wasps parasitize only a small fraction of the aphids and do not compete significantly with the ants for honeydew. Low rates of oviposition in small aphid colonies could be favored by selection if high rates of parasitism led to a risk of ant abandonment prior to parasitoid progeny emergence, and thus reduced female fitness relative to a more conservative strategy of host parasitism within patches.
